# Identification of Endogenous Reference Genes for the Analysis of microRNA Expression in the Hippocampus of the Pilocarpine-Induced Model of Mesial Temporal Lobe Epilepsy

**DOI:** 10.1371/journal.pone.0100529

**Published:** 2014-06-25

**Authors:** Mykaella Andrade de Araújo, Thalita Ewellyn Batista Sales Marques, Jamile Taniele-Silva, Fernanda Maria de Araújo Souza, Tiago Gomes de Andrade, Norberto Garcia-Cairasco, Maria Luisa Paçó-Larson, Daniel Leite Góes Gitaí

**Affiliations:** 1 Department of Cell, Molecular Biology, Institute of Biological Sciences and Health, Federal University of Alagoas, Maceió, Alagoas, Brazil; 2 Campus Arapiraca, Federal University of Alagoas, Maceió, Alagoas, Brazil; 3 Department of Physiology, Ribeirão Preto School of Medicine, University of São Paulo, Ribeirão Preto, São Paulo, Brazil; 4 Department of Cellular and Molecular Biology, Ribeirão Preto School of Medicine, University of São Paulo, Ribeirão Preto, São Paulo, Brazil; University Paris 6, France

## Abstract

Real-time quantitative RT-PCR (qPCR) is one of the most powerful techniques for analyzing miRNA expression because of its sensitivity and specificity. However, in this type of analysis, a suitable normalizer is required to ensure that gene expression is unaffected by the experimental condition. To the best of our knowledge, there are no reported studies that performed a detailed identification and validation of suitable reference genes for miRNA qPCR during the epileptogenic process. Here, using a pilocarpine (PILO) model of mesial temporal lobe epilepsy (MTLE), we investigated five potential reference genes, performing a stability expression analysis using geNorm and NormFinder softwares. As a validation strategy, we used each one of the candidate reference genes to measure PILO-induced changes in microRNA-146a levels, a gene whose expression pattern variation in the PILO injected model is known. Our results indicated U6SnRNA and SnoRNA as the most stable candidate reference genes. By geNorm analysis, the normalization factor should preferably contain at least two of the best candidate reference genes (snoRNA and U6SnRNA). In fact, when normalized using the best combination of reference genes, microRNA-146a transcripts were found to be significantly increased in chronic stage, which is consistent with the pattern reported in different models. Conversely, when reference genes were individually employed for normalization, we failed to detect up-regulation of the microRNA-146a gene in the hippocampus of epileptic rats. The data presented here support that the combination of snoRNA and U6SnRNA was the minimum necessary for an accurate normalization of gene expression at the different stages of epileptogenesis that we tested.

## Introduction

Temporal lobe epilepsy (TLE) is one of the most common medically intractable neurological disorders. The pathogenesis of TLE involves abnormal neuronal reorganization occurring over a long period of time following a strong cerebral insult, such as status epilepticus (SE) [1], [2]. These changes can include neurodegeneration, neurogenesis, gliosis, axonal damage or sprouting, dendritic plasticity, inflammation, reorganization of the extracellular matrix and alterations in the molecular structure of cellular components [3]. Evidence is emerging that these processes could be associated with network-wide changes of protein-coding transcript levels. In fact, studies in patients or animal models of TLE show that the global gene expression pattern is significantly altered at time points more closely related to responses to either SE or cumulative chronic spontaneous recurrent seizures (SRS) [4]–[9]. Therefore, epilepsy research has turned to the question of which regulator factors are involved in the reorganization of gene expression that accompanies the epileptic condition.

MicroRNAs (miRNA) represent a family of small (22–24 nucleotides), endogenous noncoding RNAs that act as small regulatory molecules involved in posttranscriptional gene repression [10]–[16]. miRNAs are involved in numerous physiological processes and increasing evidence suggest that miRNAs are deregulated in several neurological diseases [17]–[21]. Recently, several miRNAs have been found to be differentially expressed in TLE models [22]–[27] encouraging further studies with this approach. Indeed, establishing reliable profiles of miRNA expression in epileptogenesis could be a significant step forward to the understanding of the roles played by these molecules in epileptogenesis.

Real-time quantitative RT-PCR (RT-qPCR) is one of the most powerful techniques for analyzing miRNA expression because of its sensitivity and specificity [28]–[30]. However, in this type of analysis, the impact of experimental variations, such as pipetting errors, reverse transcription efficiency, qPCR cycling conditions, RNA quality and purity, the stability and heterogeneity of the microRNAs in the sample, age-unmatching of experimental individuals with controls, has to be considered [31], [32]. Also, a suitable normalizer is required to remove as much variation as possible leading to an increase of the accuracy of expression measurements [33]. The impact of using an unstable internal control can lead to inaccurate results and erroneous conclusions. It is essential, therefore, to identify and validate the reference genes prior to their use for normalization in specific experimental set ups.

In previous study, we reported suitable reference genes for mRNA qPCR in two different model of TLE [34]. However, they may be not suitable internal controls for miRNA qPCR analysis. In fact, miRNAs pose a significant challenge for normalization, because they represent perhaps only 0.01% of the mass of total RNA in a sample, and this fraction can vary significantly across different samples [35]. Moreover, the extraction efficiency of miRNA from samples is very different than for much longer RNAs. Some studies have used synthetic miRNA molecules as reference genes [36], despite of the fact that they cannot correct sample-to-sample variation. In epileptic conditions, small RNA molecules have also been used as reference genes, however without a preliminary and systematic evaluation of their suitability [37], [38]. Particularly, for the analysis of the expression of mir146 (used here as target gene) in the epileptogenic process, the U6snRNA has been used as a reference gene, also, without previous validation [22], [39]–[42].

Therefore, to investigate the expression patterns of miRNAs during epileptogenesis, suitable internal controls for miRNA qPCR need to be identified. Here, using a pilocarpine (PILO) model of temporal lobe epilepsy (TLE), we investigated five potential reference genes recommended by a leading commercial miRNA assay supplier (Applied Biosystems, 2007), including U6SnRNA (001973 Assay ID), SnoRNA (001718 Assay ID), Y1 (001727 Assay ID), 4.5(S)1 (001716 Assay ID) and 4.5(S)5 (001717 Assay ID). The gene expression levels were investigated by qPCR in the hippocampus of experimental and control animals, followed by a stability expression analysis using geNorm and NormFinder softwares. Finally, as a validation strategy, we used each one of the candidate reference genes to measure PILO-induced changes in miRNA-146a, a gene whose expression pattern variation in PILO-injected model is known [42]. In fact, mir146 is overexpressed in all epilepsy associated condictions tested, including PILO and electrical stimulation TLE models and human TLE with hippocampal sclerosis [22],[39]–[42].

## Results

### Analysis of candidate reference genes for microRNA qRT-PCR in PILO-induced model of epilepsy

**Figure 1:**
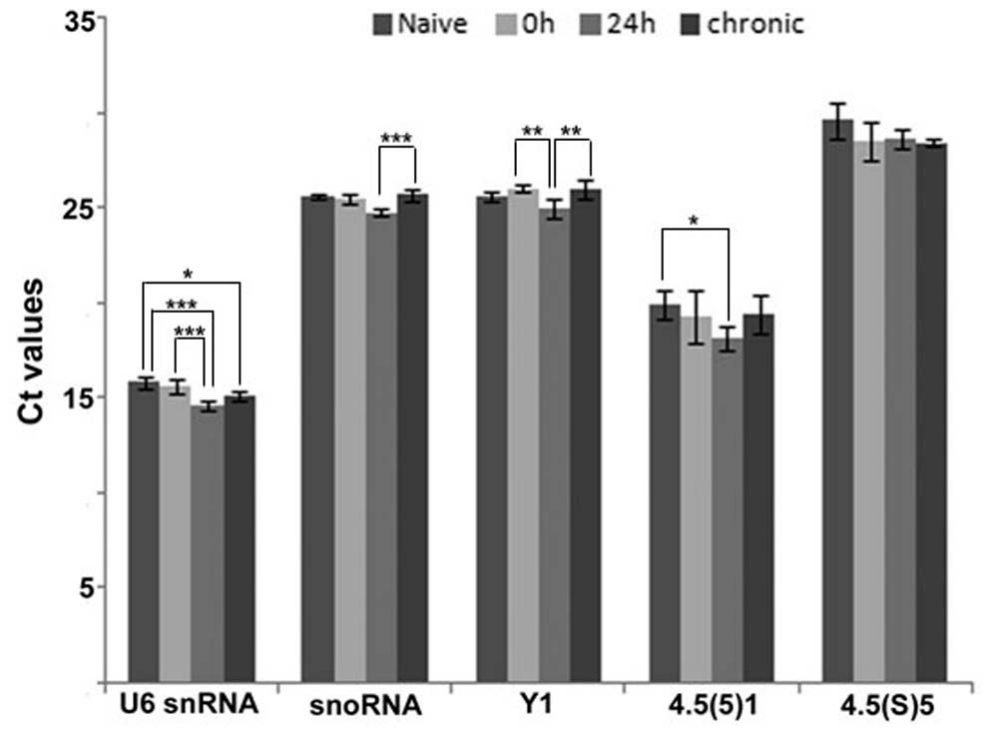
Levels of five non-coding RNAs candidate reference genes for microRNA qRT-PCR analysis in the hippocampus of the systemic PILO-injected and control rats. Values are given in the form of RT-qPCR threshold cycle numbers (Ct values), mean 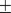 SD (n = 6). ANOVA, * 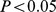, ** 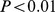, *** 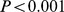.

Our main objective was to identify small RNAs which could be used as reference genes in qRT-PCR analysis of hippocampus samples of rats submitted to systemic PILO injection and naive animals. Figure 1 gives the mean of Ct (cycle threshold) values for each one of the five candidate reference genes analyzed, illustrating the levels of these RNAs among the different experimental groups. The analyzed genes displayed a relatively wide range of expression, with mean Ct values between 14.58 (U6SnRNA) and 29.57 (4.5(S)5). Also, with exception of 4.5(S)5, we observed differences in the average levels among the experimental groups.

Therefore, in order to determine the least variable reference genes in systemic-PILO-injected model, we evaluated expression stability of the five candidate controls in our sample from different periods of the epileptogenic process, using geNorm and Normfinder softwares.

**Figure 2:**
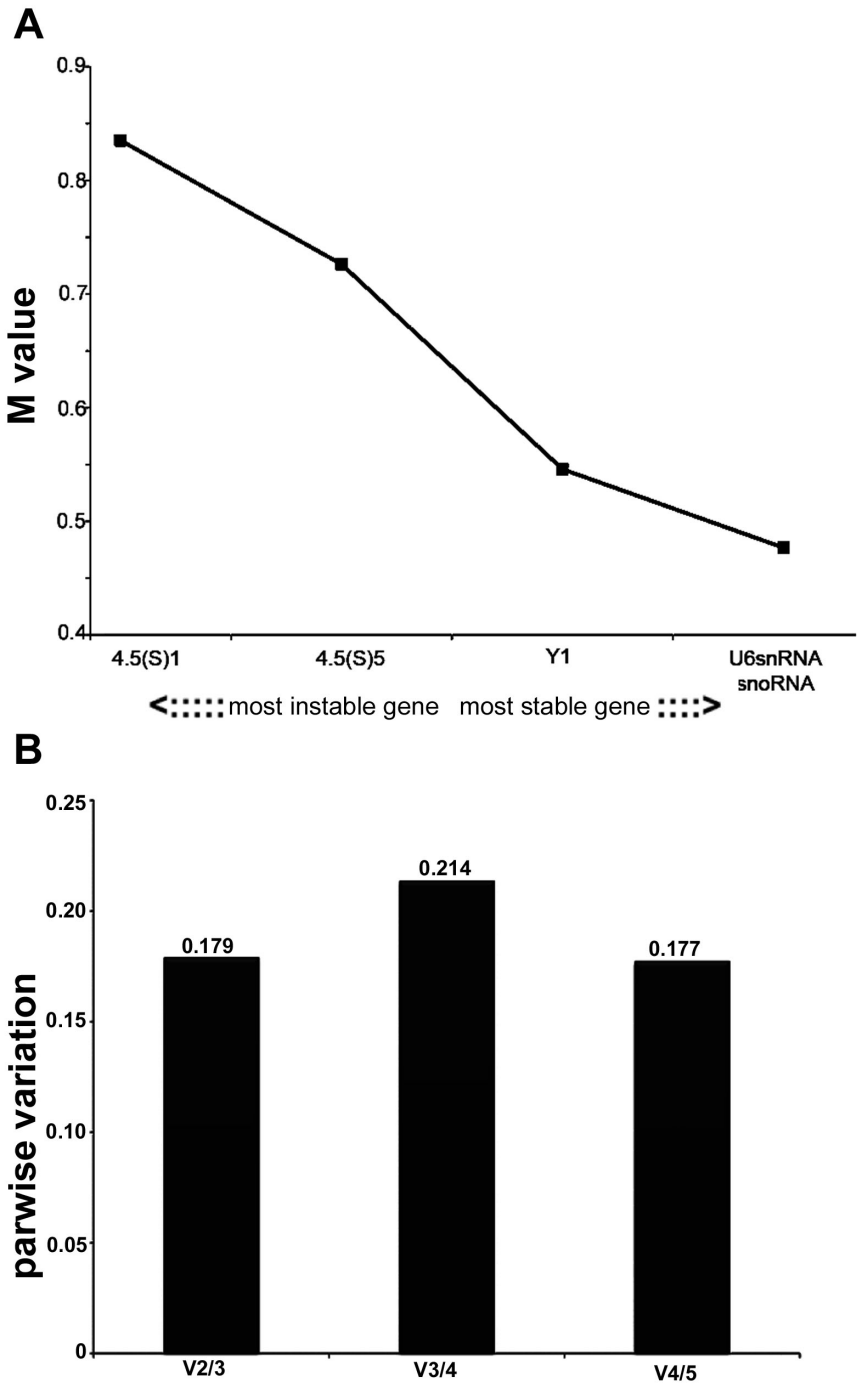
Selection of the most suitable reference genes for microRNA qRT-PCR analysis in the systemic PILO-model samples using geNorm analysis. A) Expression stability measurements (M) for the five reference genes analyzed. The x-axis from left to right indicates the ranking of the genes according to their expression stability; lower M values indicate higher expression stability. B) Determination of the optimal number of reference genes for normalization was conducted. The software calculates the normalization factor from at least two genes at which the variable V defines the pair-wise variation between two sequential normalization factors.

geNorm calculates a gene-stability measure (M) based on the average pairwise variation between a particular gene and all other studied genes. High expression stability is indicated by a low M value as an estimate of combined variation of the individual gene. Successive elimination of the least stable gene ranks the candidate housekeeping genes according to their M values and identifies the two most stable reference genes [43]. The average expression stability values (M values) of the analyzed genes in all tested samples from systemic PILO-injected rats are displayed in Figure 2A. From the most stable to the least stable, the genes were ranked as follows: snoRNA, U6SnRNA, Y1, 4.5(s)5 and 4.5(s)1, being snoRNA and U6SnRNA selected as the best combination of two genes.

To determine the minimum number of reference genes necessary for an accurate normalization, a pairwise variation Vn/Vn+1 analysis was performed (Figure 2B) [43]. Here, the V2/3 value was 0.179 which was nearest to the default cutoff value. Therefore, the normalization factor should preferably contain at least two of the best candidate reference genes (snoRNA and U6SnRNA).

**Figure 3:**
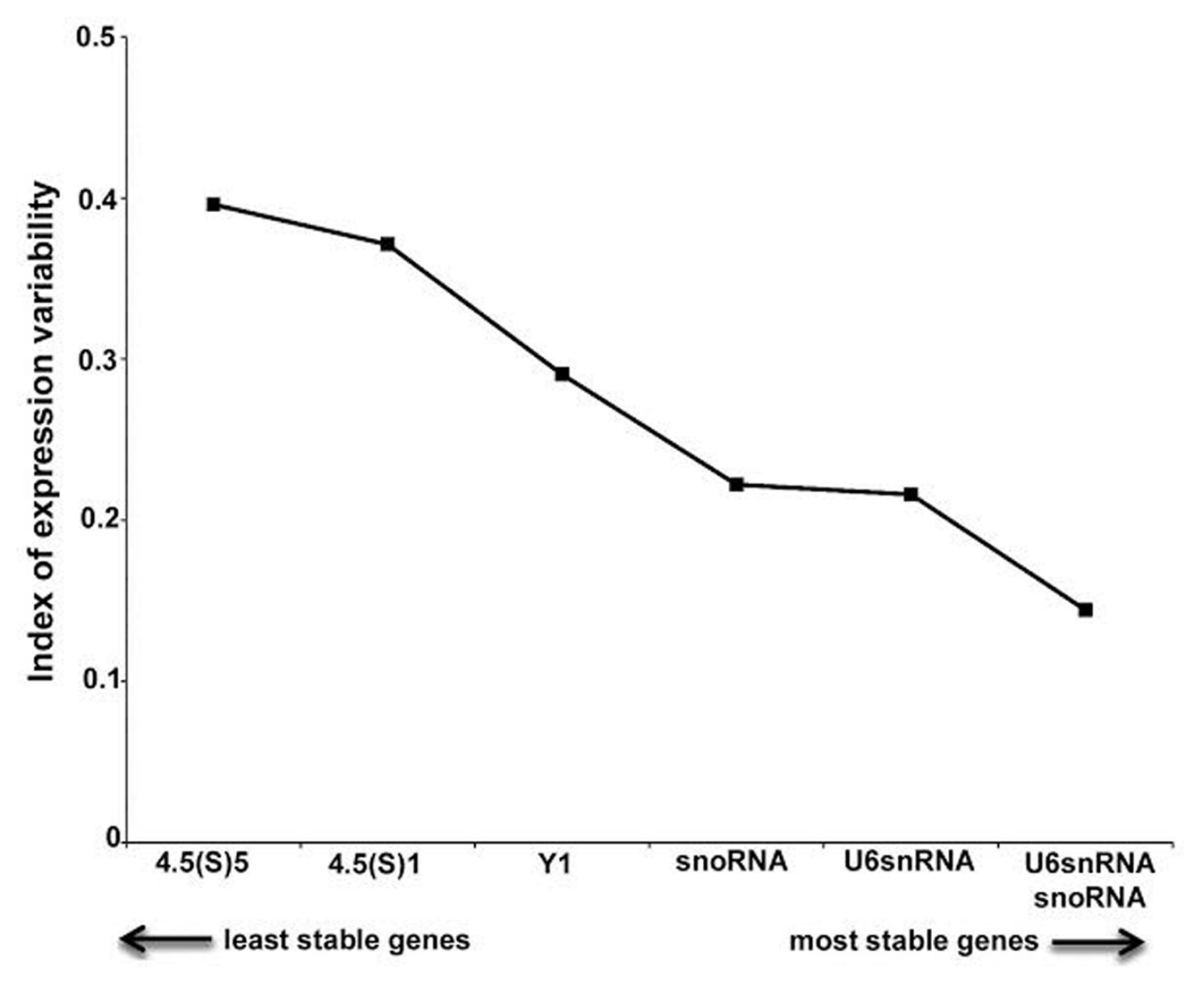
NormFinder analysis of expression stability of candidate reference genes for microRNA qRT-PCR analysis in the systemic PILO-model samples. Ranking of candidate reference genes based on stability values calculated by NormFinder software.

Expression stability of snoRNA, U6SnRNA, Y1, 4.5(s)5 and 4.5(s)1 RNAs was additionally evaluated with NormFinder, another software that uses a model-based approach to measure the variation in gene expression among sample subgroups [44]. NormFinder calculates stability values for each analyzed gene on the basis of inter- and intra-group expression variation. The lower stability values indicate the more stable expressed candidate genes. Results of NormFinder analysis are shown in Figure 3. U6SnRNA, snoRNA, Y1, 4.5(s) variant 1 and 4.5(s) variant 5 appeared as the most stable genes (stability between 0.216 and 0.396). The best combination of reference genes indicated was also snoRNA and U6SnRNA. These data sets are comparable with those obtained using geNorm, with slight differences in the ranking order.

### Validation of the reference genes

**Figure 4:**
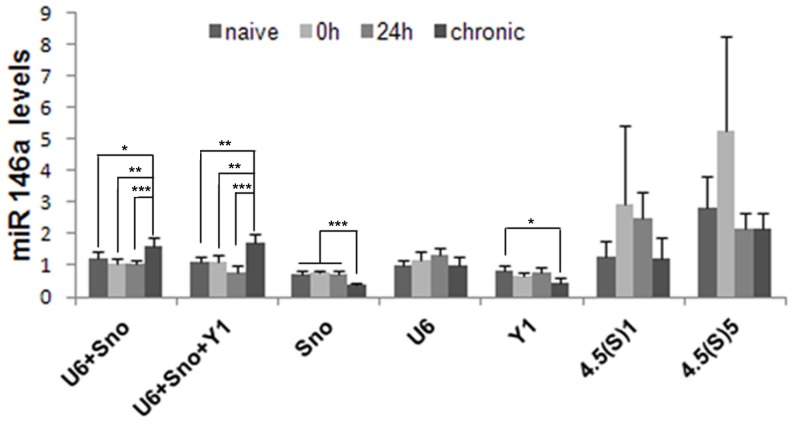
Relative quantities of miR-146a in the hippocampus of the systemic PILO- injected rats upon different normalization approaches. qRT-PCR data were normalized by single reference gene and best combination derived by geNorm or NormFinder analysis (mean 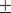 SD, n = 6). The diagram shows mean levels of miR-146a transcripts in naive animals, epileptogenesis (0 h and 24 h) and chronic period. ANOVA, * 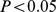, ** 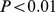, *** 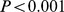.

In order to validate the results obtained, we conducted a relative expression analysis of the miRNA-146a gene, whose mRNA expression pattern in the hippocampus of animal models of TLE is known [22], [25], [42], [45], comparing all experimental and control groups. We used each of the five reference genes as internal controls, as well as the combination of three or two genes, recommended by the analysis using both geNorm and NormFinder (Figure 4).

Using the best combination of three reference genes for normalization, miRNA-146a transcripts were found to be significantly increased in the chronic stage, which is consistent with the pattern reported in different models. Similar expression patterns were generated when either two of the most stable genes were used for normalization. Conversely, when reference genes were individually employed for normalization, we failed to detect upregulation of miRNA-146a in the hippocampus of epileptic rats.

## Discussion

Since small changes in the expression of a single miRNA may affect multiple genes, it is not unexpected that accurate measurement of miRNA expression is a critical requisite [33], [46]. Studies that aim to detect subtle changes in miRNA regulation, particularly using qPCR, must use proper reference genes to avoid erroneous conclusions. Currently, the majority of the studies investigating miRNA expression in animal models of TLE have used reference genes without systematic validation of their stability. In fact, to the best of our knowledge, this is the first report detailing the identification and validation of suitable reference genes for miRNA qPCR assay, despite the surge of interest in miRNA identification and quantification during the epileptogenic process.

Ideally, reference genes should present high expression stability levels in different experimental conditions [47], [48]. The evaluation of a panel of five candidate reference genes to determine the most reliable one for accurate normalization of gene expression in the systemic PILO-model indicated two (snoRNA and U6SnRNA) as the most stable in the hippocampus of rats from all experimental and control groups. Depending on the software used (geNorm or NormFinder), the rank of these genes on a stability scale was slightly different, probably because of the different mathematical algorithm employed [44], [43].

We also considered whether selecting multiple reference genes in combination is better than selecting a single reference gene alone. The optimal number of reference genes which should be used for accurate normalization was determined by calculating the normalization factor (NF). The use of more than the two most stable reference genes identified (snoRNA/U6SnRNA) is not required as suggested by the V-value (Figure 3).

In order to evaluate the functional significance of the results obtained for reference genes, we conducted a relative expression analysis of the miR-146a gene, whose pattern is already described for the PILO-injected model. miR-146a can be induced by different pro-inflammatory stimuli and has been shown to be upregulated in experimental models of epilepsy, as well as in human TLE. Indeed, in PILO and electrical stimulation TLE models, prominent upregulation of miRNA-146a was evident at 1-3 week after SE and persisted in the chronic phase [22], [39]–[42]. In human TLE with hippocampal sclerosis, increased astroglial expression of miR-146a was observed mainly in regions where neuronal cell loss and reactive gliosis occurred [22]. Similarly, Iyer *et al.* [45] showed an overexpression of mir-146a in epilepsy-associated glioneuronal lesions. It remains unclear how the induction of mir146 expression may contribute to the etiopathogenesis of TLE. Thus, Iyer *et al.* [45] observed that seizures alone may not account for changes in miR-146a expression. Moreover, emerging data suggest that miR-146a is induced as a negative-feedback regulator of the glial-mediated inflammatory response associated to the epileptogenic process [22], [42], [45]. This is in line with other studies supporting an immunomodulatory role ascribed to miR-146a in several human neurodegenerative diseases associated with a strong inflammatory component [49], [50]. In fact, upregulation of miR146a has been detected in active multiple sclerosis lesions [51], in human Alzheimer disease (AD) brain [52] and in prion disease [50], [53], indicating an underlying common inflammatory response to different neurological insults. Accordingly, when normalized using the best combination of two or three reference genes, we observed that miR-146a transcript levels were significantly increased in the chronic stage. Interestingly, under our experimental conditions, the use of a reference gene individually for normalization leads to the relative transcript levels of the mir-146a gene to be different from those obtained with the best combinations of genes, and hence probably less accurate. This suggests, therefore, that an appropriate normalization strategy for miRNA expression during epileptogenesis requires the use of two or more reference genes.

Curiously, in previous studies, under the same experimental conditions, in order to normalize protein-coding RNA expression, the use of just one of the stable reference genes produced a reliable measurement [34]. These differences could be explained by the use of different methods for sample processing, including RNA extraction and cDNA synthesis. For instance, in order for the specific detection of mature mirs using TaqMan MicroRNA assays (Applied Biosystems, Foster City, CA), the reverse transcription step is performed separately for each gene, which may result in higher methodological variations. This is supported here by the comparative analysis of the raw Ct values, which showed for all candidate reference genes significant differences among experimental groups, with an exception for 4.5(S)5. Some authors also observed that the normalization factor for analysis of miRNA expression should preferably contain at least three of the best candidate reference genes; also non-coding RNAs presented better expression stability than protein coding RNAs [54]. However, this cannot be inferred to be true for other experimental conditions. In fact, four independent studies using RT-qPCR showed the up-regulation of miR-146a in the hippocampus of rat and human with epilepsy, even using a normalization approach based on only one reference gene (U6snRNA) and without prior stability analysis [22], [40]–[42]. In our study, the U6snRNA had the most stable expression levels during epileptogenesis as pointed out by NormFinder analysis. However, the normalization with U6SnRNA alone leads to the increased expression profile without significance. These findings emphasize that a normalization strategy for a pathology model may not be appropriate in a different experimental condition related to the same pathology.

Finally, we recognize that the effort, cost, and sample requirements necessary for the experimental selection of miRNA normalizers is not always possible. In such cases, the data presented here suggests that the combination of snoRNA and U6SnRNA is likely a more reasonable choice than using a randomly selected reference gene for the investigation of epileptogenesis.

## Materials and Methods

### Animals

Experiments were conducted in Wistar male rats (n = 42) from the main breeding stock of the Federal University of Alagoas. All rats were 90-100-days-old and weighted from 200 to 250 g. They were kept at 22°C in groups of four per cage with free access to food and water, in a 12 h light/dark cycle (lights on at 08:00). All experimental procedures were performed according to the Brazilian Society for Neuroscience and Behavior, which are based on international guidelines of the ethical use of animals, such as those from the Society for Neuroscience. The protocols were approved by the Research Ethics Committee of the Federal University of Alagoas (Permit number: 01146a2/2010-83). All efforts were made to minimize the number of animals used and to avoid any unnecessary suffering.

### PILO injection

Animals were injected intra-peritoneally with scopolamine butyl bromide (1 mg/kg; ip) in order to reduce peripheral cholinergic effects, followed by PILO (ip) in a dose of 320 mg/kg after 30 min. All animals that had SE were rescued with diazepam (DZP) (5 mg/kg; ip), 90 min after SE establishment. Out of 36 PILO-injected rats, 18 died during the experiments, and 18 developed SE. From the third day after SE, animals (chronic group) were individually placed in acrylic cages and their behavior was recorded on videotapes for up to 6 hours per day. Three groups of rats subjected to SE were used: animals sacrificed immediately (n = 6), twenty four hours (n = 6) and 9–14 weeks after SE (n = 6). All animals from this last group showed two or more SRS with seizure severity scores equal or greater than 3, according to the scale of Racine [55]. Naive rats were used as control group (n = 6).

### RNA extraction and RT-qPCR

Rats were guillotined and the brains were immediately dissected on ice. Hippocampi were rapidly frozen and stored in liquid nitrogen until RNA isolation. Total RNA was purified using Trizol reagent (Invitrogen, CA, USA), following the manufacturers protocol. The quality of total RNA was assessed by analysis of the ratio of 28S to 18S ribosomal RNAs after electrophoresis in 1% agarose gel. miRNA expression was analyzed using Taqman microRNA assays (Applied Biosystems, Foster City, CA). Gene-specific reverse transcription (RT) for miR and snRNAs was performed using 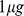 of purified total RNA, 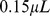 of 100 mM dNTPs (with dTTP), 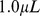 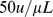 MultiScribe Reverse Transcriptase, 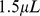 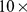 RT Buffer, 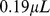 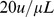 RNase Inhibitor, 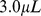 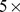 TaqMan microRNA RT Primer and 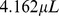 nuclease free water. Fifteen microliter reaction was incubated for 30 min at 16°C, 30 min at 42°C, and 5 min at 85°C to inactivate reverse transcriptase. Realtime PCR reaction, including 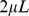 of RT product, 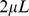 nuclease free water, 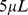 TaqMan 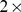 Universal PCR master mix (Applied Biosystems) and 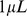 TaqMan microRNA Assay containing PCR primers and TaqMan probes, was run in triplicate on the StepOnePlus (Applied Biosystems) at 95°C for 10 min followed by 40 cycles at 95°C for 15s and 60°C for 1 min.

### Determination of reference gene expression stability

To assess the stability of candidate reference genes, two commonly used approaches, geNorm (http://medgen.ugent.br/~jvdesomp/genorm/) and NormFinder (http://www.mdl.dk/publicationsnormfinder.html) algorithms, were utilized. For this, Ct values were converted into relative quantities via the delta-Ct method using the sample with the lowest Ct as calibrator, in accordance with the 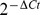 method [56].

geNorm uses an algorithm to calculate the M value, a gene expression stability factor defined as the mean pairwise variation for a given gene compared to the remaining tested genes. Hence, a lower M value indicates higher stability of the reference gene. The program also estimates the pairwise variation between two sequential calculations of normalization factors (NF) including an increasing number of genes. This defines the minimal number of genes required to calculate a robust normalization factor. NormFinder uses an ANOVA-based model to estimate intra and inter-group variation, and combines these estimates to provide a direct measure of the variation in expression for each gene.

### Reference gene validation

miRNA-146a transcripts were used as the target gene in order to validate the best reference genes for normalization of relative expression in epileptogenesis induced by PILO. Its relative quantity in each sample was normalized either to the most stable combination, in accordance with geNorm and NormFinder analyses, or to each of the five reference genes independently, using the 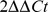 method [56].

Statistical analysis of data from different animal groups was done using the multiple comparative Tukey-Kramer test (InStat, version 3.01).

## References

[1] Mathern GW, Adelson PD, Cahan LD, Leite JP (2002) Hippocampal neuron damage in human epilepsy: Meyer's hypothesis revisited. Prog Brain Res 135: 237–251. 12143344 10.1016/s0079-6123(02)35023-4

[2] Wiebe S, Blume WT, Girvin JP, Eliasziw M (2001) A randomized, controlled trial of surgery for temporal-lobe epilepsy. N Engl J Med 345: 311–318. 11484687 10.1056/NEJM200108023450501

[3] Pitkanen A, Lukasiuk K (2009) Molecular and cellular basis of epileptogenesis in symptomatic epilepsy. Epilepsy Behav 14 Suppl 116–25. 18835369 10.1016/j.yebeh.2008.09.023

[4] Becker AJ, Chen J, Zien A, Sochivko D, Normann S, et al (2003) Correlated stage- and subfield-associated hippocampal gene expression patterns in experimental and human temporal lobe epilepsy. Eur J Neurosci 18: 2792–2802. 14656328 10.1111/j.1460-9568.2003.02993.x

[5] Elliott RC, Miles MF, Lowenstein DH (2003) Overlapping microarray profiles of dentate gyrus gene expression during development- and epilepsy-associated neurogenesis and axon outgrowth. J Neurosci 23: 2218–2227. 12657681 10.1523/JNEUROSCI.23-06-02218.2003 PMC6742005

[6] Gorter JA, van Vliet EA, Aronica E, Breit T, Rauwerda H, et al (2006) Potential new antiepileptogenic targets indicated by microarray analysis in a rat model for temporal lobe epilepsy. J Neurosci 26: 11083–11110. 17065450 10.1523/JNEUROSCI.2766-06.2006 PMC6674659

[7] Hunsberger JG, Bennett AH, Selvanayagam E, Duman RS, Newton SS (2005) Gene profiling the response to kainic acid induced seizures. Brain Res Mol Brain Res 141: 95–112. 16165245 10.1016/j.molbrainres.2005.08.005

[8] Lukasiuk K, Kontula L, Pitkanen A (2003) cDNA profiling of epileptogenesis in the rat brain. Eur J Neurosci 17: 271–279. 12542663 10.1046/j.1460-9568.2003.02461.x

[9] Okamoto OK, Janjoppi L, Bonone FM, Pansani AP, da Silva AV, et al (2010) Whole transcriptome analysis of the hippocampus: toward a molecular portrait of epileptogenesis. BMC Genomics 11: 230. 20377889 10.1186/1471-2164-11-230 PMC2859406

[10] Bartel DP (2004) MicroRNAs: genomics, biogenesis, mechanism, and function. Cell 116: 281–297. 14744438 10.1016/s0092-8674(04)00045-5

[11] Chen K, Rajewsky N (2007) The evolution of gene regulation by transcription factors and microRNAs. Nat Rev Genet 8: 93–103. 17230196 10.1038/nrg1990

[12] Djuranovic S, Nahvi A, Green R (2011) A parsimonious model for gene regulation by miRNAs. Science 331: 550–553. 21292970 10.1126/science.1191138 PMC3955125

[13] Huntzinger E, Izaurralde E (2011) Gene silencing by microRNAs: contributions of translational repression and mRNA decay. Nat Rev Genet 12: 99–110. 21245828 10.1038/nrg2936

[14] Kim VN, Han J, Siomi MC (2009) Biogenesis of small RNAs in animals. Nat Rev Mol Cell Biol 10: 126–139. 19165215 10.1038/nrm2632

[15] Krol J, Loedige I, Filipowicz W (2010) The widespread regulation of microRNA biogenesis, function and decay. Nat Rev Genet 11: 597–610. 20661255 10.1038/nrg2843

[16] Pillai RS, Bhattacharyya SN, Filipowicz W (2007) Repression of protein synthesis by miRNAs: how many mechanisms? Trends Cell Biol 17: 118–126. 17197185 10.1016/j.tcb.2006.12.007

[17] Hebert SS, De Strooper B (2009) Alterations of the microRNA network cause neurodegenerative disease. Trends Neurosci 32: 199–206. 19268374 10.1016/j.tins.2008.12.003

[18] Lukiw WJ, Alexandrov PN (2012) Regulation of complement factor H (CFH) by multiple miRNAs in Alzheimer's disease (AD) brain. Mol Neurobiol 46: 11–19. 22302353 10.1007/s12035-012-8234-4 PMC3703615

[19] Lukiw WJ, Pogue AI (2007) Induction of specific micro RNA (miRNA) species by ROS-generating metal sulfates in primary human brain cells. J Inorg Biochem 101: 1265–1269. 17629564 10.1016/j.jinorgbio.2007.06.004 PMC2080079

[20] Redell JB, Liu Y, Dash PK (2009) Traumatic brain injury alters expression of hippocampal microRNAs: potential regulators of multiple pathophysiological processes. J Neurosci Res 87: 1435–1448. 19021292 10.1002/jnr.21945 PMC5980641

[21] Redell JB, Zhao J, Dash PK (2011) Altered expression of miRNA-21 and its targets in the hippocampus after traumatic brain injury. J Neurosci Res 89: 212–221. 21162128 10.1002/jnr.22539 PMC7958494

[22] Aronica E, Fluiter K, Iyer A, Zurolo E, Vreijling J, et al (2010) Expression pattern of miR-146a, an inflammation-associated microRNA, in experimental and human temporal lobe epilepsy. Eur J Neurosci 31: 1100–1107. 20214679 10.1111/j.1460-9568.2010.07122.x

[23] Jimenez-Mateos EM, Engel T, Merino-Serrais P, McKiernan RC, Tanaka K, et al (2012) Silencing microRNA-134 produces neuroprotective and prolonged seizure-suppressive effects. Nat Med 18: 1087–1094. 22683779 10.1038/nm.2834 PMC3438344

[24] Risbud RM, Lee C, Porter BE (2011) Neurotrophin-3 mRNA a putative target of miR21 following status epilepticus. Brain Res 1424: 53–59. 22019057 10.1016/j.brainres.2011.09.039 PMC4410817

[25] Risbud RM, Porter BE (2013) Changes in microRNA expression in the whole hippocampus and hippocampal synaptoneurosome fraction following pilocarpine induced status epilepticus. PLoS ONE 8: e53464. 23308228 10.1371/journal.pone.0053464 PMC3538591

[26] Sano T, Reynolds JP, Jimenez-Mateos EM, Matsushima S, Taki W, et al (2012) MicroRNA-34a upregulation during seizure-induced neuronal death. Cell Death Dis 3: e287. 22436728 10.1038/cddis.2012.23 PMC3317348

[27] Song YJ, Tian XB, Zhang S, Zhang YX, Li X, et al (2011) Temporal lobe epilepsy induces differential expression of hippocampal miRNAs including let-7e and miR-23a/b. Brain Res 1387: 134–140. 21376023 10.1016/j.brainres.2011.02.073

[28] Bustin SA (2002) Quantification of mRNA using real-time reverse transcription PCR (RT-PCR): trends and problems. J Mol Endocrinol 29: 23–39. 12200227 10.1677/jme.0.0290023

[29] Cikos S, Koppel J (2009) Transformation of real-time PCR fluorescence data to target gene quantity. Anal Biochem 384: 1–10. 18812161 10.1016/j.ab.2008.08.031

[30] Ginzinger DG (2002) Gene quantification using real-time quantitative PCR: an emerging technology hits the mainstream. Exp Hematol 30: 503–512. 12063017 10.1016/s0301-472x(02)00806-8

[31] Chugh P, Dittmer DP (2012) Potential pitfalls in microRNA profiling. Wiley Interdiscip Rev RNA 3: 601–616. 22566380 10.1002/wrna.1120 PMC3597218

[32] Pritchard CC, Cheng HH, Tewari M (2012) MicroRNA profiling: approaches and considerations. Nat Rev Genet 13: 358–369. 22510765 10.1038/nrg3198 PMC4517822

[33] Bustin SA, Benes V, Nolan T, Pfaffl MW (2005) Quantitative real-time RT-PCR–a perspective. J Mol Endocrinol 34: 597–601. 15956331 10.1677/jme.1.01755

[34] Marques TE, de Mendonca LR, Pereira MG, de Andrade TG, Garcia-Cairasco N, et al (2013) Validation of suitable reference genes for expression studies in different pilocarpine-induced models of mesial temporal lobe epilepsy. PLoS ONE 8: e71892. 24009668 10.1371/journal.pone.0071892 PMC3751890

[35] Liang Y, Ridzon D, Wong L, Chen C (2007) Characterization of microRNA expression profiles in normal human tissues. BMC Genomics 8: 166. 17565689 10.1186/1471-2164-8-166 PMC1904203

[36] Kang K, Peng X, Luo J, Gou D (2012) Identification of circulating miRNA biomarkers based on global quantitative real-time PCR profiling. J Anim Sci Biotechnol 3: 4. 22958414 10.1186/2049-1891-3-4 PMC3415128

[37] Jimenez-Mateos EM, Bray I, Sanz-Rodriguez A, Engel T, McKiernan RC, et al (2011) miRNA Expression profile after status epilepticus and hippocampal neuroprotection by targeting miR-132. Am J Pathol 179: 2519–2532. 21945804 10.1016/j.ajpath.2011.07.036 PMC3204080

[38] Peng J, Omran A, Ashhab MU, Kong H, Gan N, et al (2013) Expression patterns of miR-124, miR-134, miR-132, and miR-21 in an immature rat model and children with mesial temporal lobe epilepsy. J Mol Neurosci 50: 291–297. 23315173 10.1007/s12031-013-9953-3

[39] Matos G, Scorza FA, Mazzotti DR, Guindalini C, Cavalheiro EA, et al (2014) The effects of sleep deprivation on microRNA expression in rats submitted to pilocarpine-induced status epilepticus. Prog Neuropsychopharmacol Biol Psychiatry 51: 159–165. 24530830 10.1016/j.pnpbp.2014.02.001

[40] Gorter JA, Iyer A, White I, Colzi A, van Vliet EA, et al (2014) Hippocampal subregion-specific microRNA expression during epileptogenesis in experimental temporal lobe epilepsy. Neurobiol Dis 62: 508–520. 24184920 10.1016/j.nbd.2013.10.026

[41] Hu K, Xie YY, Zhang C, Ouyang DS, Long HY, et al (2012) MicroRNA expression profile of the hippocampus in a rat model of temporal lobe epilepsy and miR-34a-targeted neuroprotection against hippocampal neurone cell apoptosis post-status epilepticus. BMC Neurosci 13: 115. 22998082 10.1186/1471-2202-13-115 PMC3471047

[42] Omran A, Peng J, Zhang C, Xiang QL, Xue J, et al (2012) Interleukin-1*β* and microRNA-146a in an immature rat model and children with mesial temporal lobe epilepsy. Epilepsia 53: 1215–1224. 22708826 10.1111/j.1528-1167.2012.03540.x

[43] Vandesompele J, De Preter K, Pattyn F, Poppe B, Van Roy N, et al (2002) Accurate normalization of real-time quantitative RT-PCR data by geometric averaging of multiple internal control genes. Genome Biol 3 RESEARCH0034. 10.1186/gb-2002-3-7-research0034 PMC126239 12184808

[44] Andersen CL, Jensen JL, Ørntoft TF (2004) Normalization of real-time quantitative reverse transcription-PCR data: a model-based variance estimation approach to identify genes suited for normalization, applied to bladder and colon cancer data sets. Cancer Res 64: 5245–5250. 15289330 10.1158/0008-5472.CAN-04-0496

[45] Iyer A, Zurolo E, Prabowo A, Fluiter K, Spliet WG, et al (2012) MicroRNA-146a: a key regulator of astrocyte-mediated inflammatory response. PLoS ONE 7: e44789. 23028621 10.1371/journal.pone.0044789 PMC3441440

[46] Peltier HJ, Latham GJ (2008) Normalization of microRNA expression levels in quantitative RT-PCR assays: identification of suitable reference RNA targets in normal and cancerous human solid tissues. RNA 14: 844–852. 18375788 10.1261/rna.939908 PMC2327352

[47] Bustin SA (2000) Absolute quantification of mRNA using real-time reverse transcription polymerase chain reaction assays. J Mol Endocrinol 25: 169–193. 11013345 10.1677/jme.0.0250169

[48] Suzuki T, Higgins PJ, Crawford DR (2000) Control selection for RNA quantitation. BioTechniques 29: 332–337. 10948434 10.2144/00292rv02

[49] Hill JM, Zhao Y, Clement C, Neumann DM, Lukiw WJ (2009) HSV-1 infection of human brain cells induces miRNA-146a and Alzheimer-type inflammatory signaling. Neuroreport 20: 1500–1505. 19801956 10.1097/WNR.0b013e3283329c05 PMC2872932

[50] Lukiw WJ, Dua P, Pogue AI, Eicken C, Hill JM (2011) Upregulation of micro RNA-146a (miRNA-146a), a marker for inflammatory neurodegeneration, in sporadic Creutzfeldt-Jakob disease (sCJD) and Gerstmann-Straussler-Scheinker (GSS) syndrome. J Toxicol Environ Health Part A 74: 1460–1468. 22043907 10.1080/15287394.2011.618973 PMC3719866

[51] Junker A, Krumbholz M, Eisele S, Mohan H, Augstein F, et al (2009) MicroRNA profiling of multiple sclerosis lesions identifies modulators of the regulatory protein CD47. Brain 132: 3342–3352. 19952055 10.1093/brain/awp300

[52] Cui JG, Li YY, Zhao Y, Bhattacharjee S, Lukiw WJ (2010) Differential regulation of interleukin-1 receptor-associated kinase-1 (IRAK-1) and IRAK-2 by microRNA-146a and NF-kappaB in stressed human astroglial cells and in Alzheimer disease. J Biol Chem 285: 38951–38960. 20937840 10.1074/jbc.M110.178848 PMC2998119

[53] Saba R, Gushue S, Huzarewich RL, Manguiat K, Medina S, et al (2012) MicroRNA 146a (miR-146a) is over-expressed during prion disease and modulates the innate immune response and the microglial activation state. PLoS ONE 7: e30832. 22363497 10.1371/journal.pone.0030832 PMC3281888

[54] Lin YL, Lai ZX (2013) Evaluation of suitable reference genes for normalization of microRNA expression by real-time reverse transcription PCR analysis during longan somatic embryogenesis. Plant Physiol Biochem 66: 20–25. 23454294 10.1016/j.plaphy.2013.02.002

[55] Racine RJ (1972) Modification of seizure activity by electrical stimulation. II. Motor seizure. Electroencephalogr Clin Neurophysiol 32: 281–294. 4110397 10.1016/0013-4694(72)90177-0

[56] Livak KJ, Schmittgen TD (2001) Analysis of relative gene expression data using real-time quantitative PCR and the 2(-Delta Delta C(T)) Method. Methods 25: 402–408. 11846609 10.1006/meth.2001.1262

